# Supplementary material for: Perioperative mortality in low-, middle-, and high-income countries: Protocol for a multi-level meta-regression analysis
Source: PLoS One. 2024 Nov 1;19(11):e0288888. doi: 10.1371/journal.pone.0288888 (PMC11530051; doi:10.1371/journal.pone.0288888)
Supplement: S1 File — (DOCX) [file pone.0288888.s001.docx]

**Supplemental File 1: Search Strategy**

**Embase Search Strategy**

Bellwether Search Strategy v13.0

Created in consultation with Jessica Moodie, MLIS

Database: Embase Classic+Embase 1947 to 2021 October 29

Date run November 1, 2021

| # | Search String | Results |
| --- | --- | --- |
| 1 | bellwether.mp. | 156 |
| 2 | open fracture/su [Surgery] | 3309 |
| 3 | ((open or compound) adj3 fracture* adj5 (surg* or reduction)).ti,ab. | 2419 |
| 4 | (open adj2 reduction).ti,ab. | 15806 |
| 5 | (c?esar?an* or csection* or c-section*).ti,ab. | 104575 |
| 6 | general surgery/ | 19159 |
| 7 | *abdominal surgery/ | 12022 |
| 8 | (abdom* adj3 (surgery or surgical or surgeries or surgically or postsurg* or post-surg* or perisurg* or peri-surg* or operation* or operating or operativ* or operated or intraoperat* or intra-operat* or postoperat* or post-operat* or perioperat* or peri-operat*)).ti,ab. | 50899 |
| 9 | ((suture* or epiploplast* or omentoplast*) adj5 (perforat* adj3 ulcer*)).ti,ab. | 211 |
| 10 | (small adj bowel* adj3 (resection* or anastomos*)).ti,ab. | 4593 |
| 11 | (colostom* or colectom* or hemicolectom*).ti,ab. | 41879 |
| 12 | (laparotom* or duodenectom* or jejunectom* or ileectom* or appendicectom* or cholecystectom* or minilaparotom* or mini-laparotom*).ti,ab. | 126045 |
| 13 | (gastroscop* adj2 (intrasurgical* or intraoperative*)).ti,ab. | 80 |
| 14 | (peritoneal adj3 (adhesionlysis or lavage)).ti,ab. | 4255 |
| 15 | (inguinal adj herniorrhaph*).ti,ab. | 1198 |
| 16 | (inguinal adj3 hernia* adj3 (repair* or surgery or surgical or surgeries or surgically)).ti,ab. | 8803 |
| 17 | (suture* adj3 (gastric adj2 (tear* or injur* or perforat*))).ti,ab. | 31 |
| 18 | or/1-17 | 365880 |
| 19 | ((an?esth* or peri-an?esth* or perian?esth* or post-an?esth* or postan?esth* or surgery or surgical or surgeries or surgically or postsurg* or post-surg* or perisurg* or peri-surg* or operation* or operating or operativ* or operated or intraoperat* or intra-operat* or postoperat* or post-operat* or perioperat* or peri-operat* or theatre or hospital or inhospital) and (death* or mortalit* or fatal* or expir* or died)).mp. | 1242547 |
| 20 | pomr.mp. | 149 |
| 21 | or/19-20 | 1242644 |
| 22 | and/18,21 | 62988 |
| 23 | (exp animal model/ or animals/) not human/ | 2540222 |
| 24 | ((animal or animals or canine* or dog or dogs or feline* or hamster* or lamb or lambs or mice or mouse or monkey or monkeys or murine or pig or pigs or porcine or piglet* or primate* or rabbit* or rat or rats or rodent* or sheep or veterinary*) not (human or patient*)).ti,kw,sh. | 5021818 |
| 25 | or/23-24 | 5159723 |
| 26 | 22 not 25 | 61468 |
| 27 | limit 26 to yr="2014 -Current" | 25166 |

**MEDLINE search strategy**

Database: Ovid MEDLINE(R) ALL 1946 to Octboer 29, 2021

Date run: November 1, 2021

| # | Search String | Results |
| --- | --- | --- |
| 1 | bellwether.mp. | 136 |
| 2 | Fractures, Open/su [Surgery] | 3967 |
| 3 | ((open or compound) adj3 fracture* adj5 (surg* or reduction)).ti,ab. | 2011 |
| 4 | (open adj2 reduction).ti,ab. | 13360 |
| 5 | (c?esar?an* or csection* or c-section*).ti,ab. | 67622 |
| 6 | General Surgery/ | 39970 |
| 7 | (abdom* adj3 (surgery or surgical or surgeries or surgically or postsurg* or post-surg* or perisurg* or peri-surg* or operation* or operating or operativ* or operated or intraoperat* or intra-operat* or postoperat* or post-operat* or perioperat* or peri-operat*)).ti,ab. | 33871 |
| 8 | ((suture* or epiploplast* or omentoplast*) adj5 (perforat* adj3 ulcer*)).ti,ab. | 146 |
| 9 | ("small bowel*" adj3 (resection* or anastomos*)).ti,ab. | 2924 |
| 10 | (colostom* or colectom* or hemicolectom*).ti,ab. | 24939 |
| 11 | (laparotom* or duodenectom* or jejunectom* or ileectom* or appendicectom* or cholecystectom* or minilaparotom* or mini-laparotom*).ti,ab. | 83682 |
| 12 | (gastroscop* adj2 (intrasurgical* or intraoperative*)).ti,ab. | 44 |
| 13 | (peritoneal adj3 (adhesionlysis or lavage)).ti,ab. | 3169 |
| 14 | "inguinal herniorrhaphy".ti,ab. | 909 |
| 15 | (inguinal adj3 hernia* adj3 (repair* or surgery or surgical or surgeries or surgically)).ti,ab. | 6487 |
| 16 | (suture* adj3 (gastric adj2 (tear* or injur* or perforat*))).ti,ab. | 24 |
| 17 | or/1-16 | 268672 |
| 18 | ((an?esth* or peri-an?esth* or perian?esth* or post-an?esth* or postan?esth* or surgery or surgical or surgeries or surgically or postsurg* or post-surg* or perisurg* or peri-surg* or operation* or operating or operativ* or operated or intraoperat* or intra-operat* or postoperat* or post-operat* or perioperat* or peri-operat* or theatre or hospital or inhospital) and (death* or mortalit* or fatal* or expir* or died)).mp. | 725821 |
| 19 | pomr.mp. | 108 |
| 20 | or/18-19 | 725895 |
| 21 | and/17,20 | 38028 |
| 22 | (exp Models, Animal/ or Animals/) not Humans/ | 4877339 |
| 23 | ((animal or animals or canine* or dog or dogs or feline* or hamster* or lamb or lambs or mice or mouse or monkey or monkeys or murine or pig or pigs or porcine or piglet* or primate* or rabbit* or rat or rats or rodent* or sheep or veterinary*) not (human or patient*)).ti,kw,sh. | 6881358 |
| 24 | or/22-23 | 6899232 |
| 25 | 21 not 24 | 36647 |
| 26 | limit 25 to yr="2014 -Current" | 11612 |

**Cochrane central register of controlled trials search strategy**

Database: [Cochrane Central Register of Controlled Trials](https://www-cochranelibrary-com.proxy1.lib.uwo.ca/), Issue 9 of 12, September 2021

Date run: September 23, 2021

| # | Search String | Results |
| --- | --- | --- |
| 1 | MeSH descriptor: [Fractures, Open] this term only and with qualifier(s): [surgery - SU] | 68 |
| 2 | (((open or compound) NEAR/3 fracture*) NEAR/5 (surg* or reduction)):ti,ab,kw | 369 |
| 3 | (open NEAR/2 reduction):ti,ab,kw | 1083 |
| 4 | (cesarean* OR csection* OR c-section*):ti,ab,kw | 11918 |
| 5 | MeSH descriptor: [General Surgery] this term only | 360 |
| 6 | (abdom* NEAR/3 (surgery or surgical or surgeries or surgically or postsurg* or post-surg* or perisurg* or peri-surg* or operation* or operating or operativ* or operated or intraoperat* or intra-operat* or postoperat* or post-operat* or perioperat* or peri-operat*)):ti,ab,kw | 10335 |
| 7 | ((suture* or epiploplast* or omentoplast*) NEAR/5 (perforat* NEAR/3 ulcer*)):ti,ab,kw | 4 |
| 8 | ("small bowel*" NEAR/3 (resection* or anastomos*)):ti,ab,kw | 82 |
| 9 | (colostom* OR colectom* OR hemicolectom*):ti,ab,kw | 2247 |
| 10 | (laparotom* OR duodenectom* OR jejunectom* OR ileectom* OR appendicectom* OR cholecystectom* OR minilaparotom* OR mini-laparotom*):ti,ab,kw | 9573 |
| 11 | (peritoneal NEAR/3 (adhesionlysis or lavage)):ti,ab,kw | 214 |
| 12 | ("inguinal herniorrhaphy"):ti,ab,kw | 297 |
| 13 | ((inguinal NEAR/5 (repair* or surgery or surgical or surgeries or surgically))):ti,ab,kw | 2851 |
| 14 | (gastric NEAR/2 (tear* OR injur* OR perforat*)):ti,ab | 215 |
| 15 | {OR #1-#14} | 36844 |
| 16 | ((an?esth* OR peri-an?esth* OR perian?esth* OR post-an?esth* OR postan?esth* OR surgery OR surgical OR surgeries OR surgically OR postsurg* OR post-surg* OR perisurg* OR peri-surg* OR operation* OR operating OR operativ* OR operated OR intraoperat* OR intra-operat* OR postoperat* OR post-operat* OR perioperat* OR peri-operat* OR theatre OR hospital OR inhospital) AND (death* OR mortalit* OR fatal* OR expir* OR died)):ti,ab,kw | 65445 |
| 17 | (pomr):ti,ab,kw | 3 |
| 18 | {OR #15-#16} | 65446 |
| 19 | {AND #14, #17} | 3824 |
| 20 | {AND #14, #17} with Publication Year from 2014 to 2021, in Trials | 1786 |

**Global index medicus search strategy**

Database: Global Index Medicus (https://www.globalindexmedicus.net/)

Date run: September 23, 2021

| # | Search String | Results |
| --- | --- | --- |
| 1 | (tw:(bellwether OR fracture* OR cesarean* OR caesarean* OR cesarian* OR caesarian* OR "general surgery" OR "general surgeries" OR abdom* OR laparotom*) AND ((ti:(anesth* OR anaesth* OR perianesth* OR perianaesth* OR postanaesth* OR postanesth* OR surgery OR surgical OR surgeries OR surgically OR postsurg* OR perisurg* OR operation* OR operating OR operativ* OR operated OR intraoperat* OR postoperat* OR perioperat* OR theatre OR hospital OR inhospital) AND (death* OR mortalit* OR fatal* OR expir* OR died)) OR (ab:(anesth* OR anaesth* OR perianesth* OR perianaesth* OR postanaesth* OR postanesth* OR surgery OR surgical OR surgeries OR surgically OR postsurg* OR perisurg* OR operation* OR operating OR operativ* OR operated OR intraoperat* OR postoperat* OR perioperat* OR theatre OR hospital OR inhospital) AND (death* OR mortalit* OR fatal* OR expir* OR died)))) AND (year_cluster:[2014 TO 2021]) | 4360 |
| Results by database: | | |
| WPRIM | | 2278 |
| LILACS | | 1159 |
| IMSEAR | | 571 |
| IMEMR | | 284 |
| AIM | |  |

**World health organization institutional repository for information sharing search strategy**

Database: World Health Organization Institutional Repository for Information Sharing (iris) (https://apps.who.int/iris/)

Date run: September 23, 2021

| # | Search String | Results |
| --- | --- | --- |
| 1 | (("open fracture" OR bellwether OR abdom* OR laparotom* OR "general surgery" OR cesarean OR caesarean OR cesarian* OR caesarian) AND (anaesth* OR anesth* OR perianesth* OR perianaesth* OR postanesth* OR postanaesth* OR surgery OR surgical OR surgeries OR surgically OR postsurg* OR perisurg* OR operation* OR operating OR operativ* OR operated OR intraoperat* OR postoperat* OR perioperat* OR theatre)) AND (death* OR mortalit* OR fatal* OR expir* OR died) | 5039 |
| Filters: | | |
| Date issued: 2014 | | 175 |
| Date issued: 2015 | | 197 |
| Date issued: 2016 | | 155 |
| Date issued: 2017 | | 163 |
| Date issued: 2018 | | 158 |
| Date issued: 2019 | | 150 |
| Date issued: 2020 | | 208 |
| Date issued: 2021 | | 110 |
| **Total** | | 1316 |
