# Supplementary material for: Perioperative mortality in low-, middle-, and high-income countries: Protocol for a multi-level meta-regression analysis
Source: PLoS One. 2024 Nov 1;19(11):e0288888. doi: 10.1371/journal.pone.0288888 (PMC11530051; doi:10.1371/journal.pone.0288888)
Supplement: S2 File — (DOCX) [file pone.0288888.s002.docx]

**Supplemental Form 2: Screening Protocol**

| This form was pilot tested on an initial search conducted September 2, 2021. One thousand results were then screened through the level one section of this form to ensure that it was capturing relevant studies.   \| Screening Questions \| Yes \| \| \| No \| \| Unclear \| \| \| \| Notes \| \| --- \| --- \| --- \| --- \| --- \| --- \| --- \| --- \| --- \| --- \| --- \| \| Title/Abstract Screening  -Judgements to be made on whether to exclude the article in question based on the following questions  -If unclear whether to exclude or include the article include at this stage \| \| \| \| \| \| \| \| \| \| \| \| 1) Are the participants living humans? \|  \| \| \|  \| \|  \| \| \| \|  \| \| If NO exclude \| \| \| \| \| \| \| \| \| \| \| \| 2) Does the study include a bellwether procedure? (caesarean section, laparotomy – see list, or treatment of open fractures – see list) that can be separated (i.e. can we get a firm number of bellwether operations and deaths within them – e.g. an arm of an RCT) \|  \| \| \|  \| \|  \| \| \| \|  \| \| If NO exclude \| \| \| \| \| \| \| \| \| \| \| \| 3) Is the study investigating children/paediatrics?  -if there is a mix between adults and children is the mean age below 18?  -if it is unreported assume it’s in adults (we will exclude at level 2 if the assumption is wrong or if it isn’t reported at all in the study) \|  \| \| \|  \| \|  \| \| \| \|  \| \| If YES exclude \| \| \| \| \| \| \| \| \| \| \| \| 4) Does the study report mortality? (saying there was no mortality is acceptable) \|  \| \| \|  \| \|  \| \| \| \|  \| \| If NO exclude \| \| \| \| \| \| \| \| \| \| \| \| 5) Does the study report POMR or are you able to calculate POMR from the reported data? (i.e. deaths/patients operated on) \|  \| \| \|  \| \|  \| \| \| \|  \| \| If NO exclude \| \| \| \| \| \| \| \| \| \| \| \| 6) Are there ≥200 participants included in the study? \|  \| \| \|  \| \|  \| \| \| \|  \| \| If NO exclude (Unless the study is from an LMIC) \| \| \| \| \| \| \| \| \| \| \| \| 7) Is the study a full paper (i.e. NOT only an abstract) \|  \| \| \|  \| \|  \| \| \| \|  \| \| If NO exclude \| \| \| \| \| \| \| \| \| \| \| \| 8) Is the study a cohort Quasi-experimental or RCT study? (e.g. exclude case reports, case series, case control*, qualitative studies, reviews and meta-analyses, editorials/opinion pieces – articles without data) \| \|  \| \| \|  \| \| \|  \| \| \| \| If NO exclude \| \| \| \| \| \| \| \| \| \| \| \| PROCEED TO FULL TEXT SCREENING FOR ARTICLES THAT HAVE NOT BEEN EXLCUDED \| \| \| \| \| \| \| \| \| \| \| \| Full Text Screening \| \| \| \| \| \| \| \| \| \| \| \| Repeat steps 1-8 to see if the full text provides more detail  -Proceed to question 9 \| \| \| \| \| \| \| \| \| \| \| \|  \| \| \| \| \| \| \| \| \| \| \|  \| \|  \| \|  \|  \| \| Screening Questions \| Yes \| \| \| No \| \| Unclear \| \| \| \| Notes \|  \|  \| \| \| If NO exclude \| \| \| \| \| \| \| \| \| \| \| \| 9) If the study is an RCT are both/all arms undergoing a surgical procedure?  If study is not an RCT skip to question 11 \|  \| \| \|  \| \|  \| \| \| \|  \|  \| \| If NO exclude ARMS of the trial that are not undergoing a surgical procedure \| \| \| \| \| \| \| \| \| \| \|  \| \|  \| \|  \|  \| \| 10) Does the study allow for POMR to be calculated from the remaining arms of the RCT? \|  \| \| \|  \| \|  \| \| \| \|  \| \| If NO exclude \| \| \| \| \| \| \| \| \| \| \|  \| \|  \| \|  \|  \| \| 11) Is the median (or mean if median is not available) age of the participants 18 years or older? \|  \| \| \|  \| \|  \| \| \| \|  \| \| If NO exclude \| \| \| \| \| \| \| \| \| \| \|  \| \|  \| \|  \|  \| \| 12) Is it possible to extract what countries the procedures/mortalities occurred in? \|  \| \| \|  \| \|  \| \| \| \|  \| \| If NO exclude \| \| \| \| \| \| \| \| \| \| \|  \| \|  \| \|  \|  \| \| 13) Is the procedure a simultaneous surgery? \|  \| \|  \| \| \| \|  \| \|  \| \|  \| \|  \| \|  \|  \| \| If YES exclude If NO INCLUDE \| \| \| \| \| \| \| \| \| \| \|  \| \|  \| \|  \|  \| |
| --- | --- | --- | --- | --- | --- | --- | --- | --- | --- | --- | --- | --- | --- | --- | --- | --- | --- | --- | --- | --- | --- | --- | --- | --- | --- | --- | --- | --- | --- | --- | --- | --- | --- | --- | --- | --- | --- | --- | --- | --- | --- | --- | --- | --- | --- | --- | --- | --- | --- | --- | --- | --- | --- | --- | --- | --- | --- | --- | --- | --- | --- | --- | --- | --- | --- | --- | --- | --- | --- | --- | --- | --- | --- | --- | --- | --- | --- | --- | --- | --- | --- | --- | --- | --- | --- | --- | --- | --- | --- | --- | --- | --- | --- | --- | --- | --- | --- | --- | --- | --- | --- | --- | --- | --- | --- | --- | --- | --- | --- | --- | --- | --- | --- | --- | --- | --- | --- | --- | --- | --- | --- | --- | --- | --- | --- | --- | --- | --- | --- | --- | --- | --- | --- | --- | --- | --- | --- | --- | --- | --- | --- | --- | --- | --- | --- | --- | --- | --- | --- | --- | --- | --- | --- | --- | --- | --- | --- | --- | --- | --- | --- | --- | --- | --- | --- | --- | --- | --- | --- | --- | --- | --- | --- | --- | --- | --- | --- | --- | --- | --- | --- | --- | --- | --- | --- | --- | --- | --- | --- | --- | --- | --- | --- | --- | --- | --- | --- | --- | --- | --- | --- | --- | --- | --- | --- | --- | --- | --- | --- | --- | --- | --- | --- | --- | --- | --- | --- | --- | --- | --- | --- | --- | --- | --- | --- | --- | --- | --- | --- | --- | --- | --- | --- | --- | --- | --- | --- | --- | --- | --- | --- | --- | --- | --- | --- | --- | --- | --- | --- | --- | --- | --- | --- | --- | --- | --- | --- | --- | --- | --- | --- | --- | --- | --- | --- | --- | --- | --- | --- | --- | --- | --- | --- | --- | --- | --- | --- | --- | --- | --- | --- | --- | --- | --- | --- | --- | --- | --- | --- | --- | --- | --- | --- | --- | --- | --- | --- | --- | --- | --- | --- | --- | --- | --- | --- | --- | --- | --- | --- | --- | --- | --- | --- | --- | --- | --- | --- | --- | --- | --- | --- | --- | --- | --- | --- | --- | --- | --- | --- | --- | --- | --- | --- | --- | --- | --- | --- | --- | --- | --- | --- | --- | --- | --- | --- | --- | --- | --- | --- | --- | --- | --- | --- | --- | --- | --- | --- | --- | --- | --- | --- | --- | --- | --- | --- | --- | --- | --- | --- | --- | --- | --- | --- | --- | --- | --- | --- | --- | --- | --- | --- | --- | --- | --- | --- | --- | --- | --- | --- | --- | --- | --- | --- | --- | --- | --- | --- | --- | --- | --- | --- | --- | --- | --- | --- | --- | --- | --- | --- | --- | --- | --- | --- | --- | --- | --- | --- | --- | --- | --- |
