# Supplementary material for: Perioperative mortality in low-, middle-, and high-income countries: Protocol for a multi-level meta-regression analysis
Source: PLoS One. 2024 Nov 1;19(11):e0288888. doi: 10.1371/journal.pone.0288888 (PMC11530051; doi:10.1371/journal.pone.0288888)
Supplement: S3 File — (DOCX) [file pone.0288888.s003.docx]

**Supplemental File 3: Exclusion Criteria Hierarchy**

| Unable to Access Article |
| --- |
| Unable to Translate |
| Abstract Only |
| Retracted Article |
| Duplicate Dataset |
| Not Investigating Perioperative Mortality |
| Wrong Intervention/ Not a Bellwether Procedure |
| Concomitant Surgery |
| Repeat Surgery |
| Wrong Patient Population/ Unable to Determine Age of Sample |
| Did Not Meet Minimum Sample Size |
| Wrong Study Design |
| Unable to Determine Number of Perioperative Mortalities |
| Aggregated Data From Multiple Countries (Contact Authors) |
