# Supplementary material for: Perioperative mortality in low-, middle-, and high-income countries: Protocol for a multi-level meta-regression analysis
Source: PLoS One. 2024 Nov 1;19(11):e0288888. doi: 10.1371/journal.pone.0288888 (PMC11530051; doi:10.1371/journal.pone.0288888)
Supplement: S4 File — (PDF) [file pone.0288888.s004.pdf]

Data Extraction

Please complete the data extraction form below for each study. If possible, at the end of each form if you could download a copy and store it in a file that may become very helpful if we have issues linking the two reviewers responses or have to do more conflict resolution.

Thank you!

If you ever cannot answer a question (e.g. the study does not provide any information regarding the question) then enter a single period (.) instead. This will tell the software that the value is missing and allow us to do analyses later to correct for that

COVIDENCE Study ID

____
(If multiple bellwethers are captured in a single study provide a record for each bellwether)

Reviewer First Name

____

Reviewer Last Name

____

Upload Study (Don't worry about this one - Kevin will upload the files)

Study Title

____

First Author Last Name

____

Year Study was Published

____

Was this study included in Ng-Kamstra 2018? (If study was published after 2015 no, otherwise check against document in Teams. bmjgh has a list of all studies included in this systematic review)

◯ Yes
◯ No
(This will be used to identify studies included since they were included by Ng-Kamstra et al 2018)

What Country was the Study Conducted In?

____

LMIC with < 200 participants.

____

Study Design

◯ Randomized Controlled Trial
◯ Prospective Cohort
◯ Retrospective Cohort
◯ Cross-Sectional
◯ Population Study/ National Database
◯ Other

What population database was used?

____

Additional Information on Study Design. Please provide more detail on the study design (e.g. interrupted time series, difference-in-difference etc)	__________________________
Bellwether Procedure(s) being studied. Please check all that apply.	☐ Cesarean Section<br>☐ Laparotomy<br>☐ Treatment of Open Fracture
Specific procedures being conducted. Please specify the specific procedures being investigated if possible.	__________________________
Hospital name. Hospital(s) where the procedures were performed.	__________________________
Level of Hospital.	○ University/Academic Hospital<br>○ Tertiary Hospital<br>○ District/Secondary Hospital<br>○ Community/Primary Hospital<br>○ Multiple Hospital Levels (e.g. multicentre studies)<br>○ Other<br>○ Unknown
More Hospital Level Information. Please provide which levels are included if from a mutlicentre study or otherwise provide further identification of hospital level.	__________________________
Year Data Collection Began	__________________________
Year Data Collection Ended	__________________________
Median Year of Data Collection	__________________________
HDI Value. Please provide the HDI value of the country that the study was conducted in at the median time of data collection.	__________________________
Is the country a High Income Country (3=yes, 0=no)	__________________________
Is the country a Middle-Income Country (2=yes, 0=no)	__________________________
Is the Country a Low-Income Country (LIC=1, ALL other countries=0)	__________________________
Summation of Groups, 3=HIC, 2=MIC, 1=LIC	__________________________

Follow-up Time. Please state how long the authors followed patients up for, in days.	◯ 28 days<br>◯ 30 days<br>◯ 42 days<br>◯ 60 days<br>◯ 90 days<br>◯ In-hospital<br>◯ Other
Please provide more detail on follow-up time	__________________
Number of Participants Included in the Study	__________________
Number of Procedures Performed.	__________________
Number of laparoscopic procedures (consider robotic surgery as laparoscopic)	__________________
Proportion of Laparoscopic Procedures	__________________
Number of deaths	__________________
POMR proportion	__________________
POMR Variance	__________________
Number of elective procedures	__________________<br>(If unclear input a single . (just a period))
Proportion of Elective Procedures	__________________
Variance of Elective Procedures	__________________
Number of People Identified as Female.	__________________
Proportion Female	__________________
Variance of proportion of females	__________________
ASA Classification	◯ Mean<br>◯ Median<br>◯ Dichotomized<br>◯ Unclear/Missing

ASA value	______________________
Age Classification	◯ Median<br>◯ Mean<br>◯ Age Category
Age Categories. Please give the age category data provided.	______________________
Age value	______________________
Location of Hospital (Urban or Rural). If the city which the hospital is located in has a population >150,000 people then it is considered urban.	◯ Unknown<br>◯ Rural<br>◯ Urban
How many people were COVID positive?	______________________
Proportion of COVID positivity.	______________________
Is this a study focusing on investigating cancer patients? (i.e. are all patients cancer patients?)	◯ No<br>◯ Yes
Does the study provide cause of death information?	◯ No<br>◯ Yes<br>◯ No deaths occurred
Please provide cause and number of deaths for each cause here. (For each cause please answer in this format: number of deaths, cause with each cause being on its own row)	______________________
Is the study focused on a high-risk group? (e.g. all emergency cases, older adults, frail people, malnutritioned etc)	◯ Yes<br>◯ No
Notes	______________________
