# Supplementary material for: Perioperative mortality in low-, middle-, and high-income countries: Protocol for a multi-level meta-regression analysis
Source: PLoS One. 2024 Nov 1;19(11):e0288888. doi: 10.1371/journal.pone.0288888 (PMC11530051; doi:10.1371/journal.pone.0288888)
Supplement: Attachment — Submitted filename: Response to Reviewers.docx [file pone.0288888.s007.docx]

Dear Dr Chen,

We would like to again thank you and the reviewers for reading our manuscript and identifying a key issue in global health meta-analysis. Below we provide a response to the reviewer’s comment.

**Reviewer #1:**  I would still recommend the authors also address/control for the publication that occurs between high vs. low income countries. The amount of data available regarding high income countries that have the funds to publish their data will always outweigh those of lower income countries will skew the data and the authors should address these issues and control for that if possible. Would DistillerSR be able to account for this bias?

**Response:** The issue of high-income countries skewing the results and masking perioperative mortality in low-income settings is a concern of ours as well. Unfortunately, DistillerSR will not be able to account for this issue. However, the problem that you have raised is the rationale for why we are conducting a multilevel analysis. This statistical technique extends the typical random effects meta-analysis to include the clustering variable of countries. By including this level, the analysis will account for clustering patterns within and between countries rather than just taking a weighted average of all the studies based upon the inverse of their variance thereby accounting for this bias. Furthermore, in the meta-regression analyses HDI will be included as a covariate in the model which should also help statistically account for this influence. In addition to these methods, we have also added an analysis of POMR stratified by income setting (ie low, middle, and high HDI groups) to ensure the data from high-income countries are not skewing the results that would be found in low resource settings using more traditional methods. Lastly, we have added a sentence to the discussion to further inform readers of this benefit regarding the use of the multilevel meta-regression analysis (lines 428-432).

Kind regards,

Kevin McIntyre
